# Four years of adolescent alcohol-related hospital treatments; trends over time

**DOI:** 10.1186/1940-0640-8-S1-A33

**Published:** 2013-09-04

**Authors:** Joris J van Hoof, Eva van Zanten, Nicolaas van der Lely

**Affiliations:** 1 Behavioral Sciences Faculty, Enschede, the Netherlands; 2 Department of Pediatrics, Reinier de Graaf Hospital, Delft, the Netherlands

## 

Alcohol intoxication among adolescents is an increasing concern in pediatric care. Alcohol use at a young age is related to alcohol abuse in later life. Also, alcohol consumption clusters with other risk seeking behaviors such as substance abuse, high risk sexual engagement, violence and aggression. The WHO recently identified alcohol use amongst young people (10-24 years) as the most important factor contributing to disability adjustable life years (DALYs). To evaluate the trends occurring in the characteristics of adolescents treated for alcohol-related harm in Dutch hospital pediatric departments from 2007 to 2010. From 2007, Dutch pediatricians (NSCK) collected data on adolescents treated for alcohol-related harm. We analyzed all data from 2007 to 2010, involving adolescents suffered from (severe) alcohol intoxication (reduced consciousness).Data was collected via a questionnaire send out to all general and academic Dutch hospitals. Children aged 11-18 years, with a BAC > 0,0 g/L and presenting with reduced consciousness were included. A total of 2023 cases were reported in the years 2007-2010 of which 1616 questionnaires were analyzed. Numbers have more than doubled over the years from 297 to 684 reports. Age is increasing among boys (14.9 to 15.6 years, P < 0.001) but not among girls (14.8 to 15.0 years, P = 0.124). Duration of reduced consciousness increased from 2.2 hrs to 3.1 hrs (P = 0.046). Boys are older (15.7 vs. 15.3 years, P < 0.001) and admitted with higher BAC than girls (1.94 vs. 1.79, P = 0.001). Boys drink more than girls during the weekend (3.34 glasses vs. 1.98 glasses, P < 0.001). Multivariate analysis shows that BAC increases with age (P = 0.042), male gender (P = 0.001) and higher educational level (P = 0.002).

